# Extensively Drug-Resistant Hypervirulent *Klebsiella pneumoniae* From a Series of Neonatal Sepsis in a Tertiary Care Hospital, India

**DOI:** 10.3389/fmed.2021.645955

**Published:** 2021-03-08

**Authors:** Tuhina Banerjee, Jayalaxmi Wangkheimayum, Swati Sharma, Ashok Kumar, Amitabha Bhattacharjee

**Affiliations:** ^1^Department of Microbiology, Institute of Medical Sciences, Banaras Hindu University, Varanasi, India; ^2^Department of Microbiology, Assam University, Silchar, India; ^3^Department of Pediatrics, Institute of Medical Sciences, Banaras Hindu University, Varanasi, India

**Keywords:** polymyxins, hypermucoviscous, MDR, carbapenems, fatal, ST5325

## Abstract

The recent emergence of multidrug-resistant (MDR) *Klebsiella pneumoniae* with hypervirulent traits causing severe infections and considerable mortality is a global cause for concern. The challenges posed by these hypermucoviscous strains of *K. pneumoniae* with regard to their optimal treatment, management, and control policies are yet to be answered. We studied a series of extensively drug-resistant (XDR) and hypervirulent *K. pneumoniae ST5235 isolates* with resistance to carbapenems and polymyxins causing neonatal sepsis in a tertiary care hospital in India. A total of 9 *K. pneumoniae* isolates from 9 cases of neonatal sepsis were studied with respect to their clinical relevance, antimicrobial susceptibility profile, presence of extended spectrum β lactamase (ESBL) production, and responsible genes, carbapenemases (classes A, B, and D), and aminoglycoside-resistant genes. Hypervirulence genes encoding hypermucoid nature, iron uptake, and siderophores were detected by multiplex PCR. The plasmid profile was studied by replicon typing. Isolates were typed by multilocus sequence typing (MLST) and enterobacterial repetitive intergenic consensus (ERIC) PCR to study the sequence types (STs) and clonal relation, respectively. The neonates in the studied cases had history of pre-maturity or low birth weight with maternal complications. All the cases were empirically treated with piperacillin–tazobactam and amikacin followed by imipenem/meropenem and vancomycin and polymyxin B as a last resort. However, all the neonates finally succumbed to the condition (100%). The studied isolates were XDR including resistance to polymyxins harboring multiple ESBL genes and carbapenemase genes (*bla*_*NDM*_ and *bla*_*OXA*−48_). Hypervirulence genes were present in various combinations with *rmpA/A2* genes present in all the isolates. IncFI plasmids were detected in these isolates. All belonged to ST5235. In ERIC PCR, 6 different clusters were seen. The study highlighted the emergence and burden of XDR hypervirulent isolates of *K. pneumoniae* causing neonatal sepsis in a tertiary care hospital.

## Introduction

Drug resistance in *Klebsiella pneumoniae* is ever increasing with adoption of several evolutionary routes by this “World Health Organization (WHO) critical priority pathogen” (1). With tremendous ability to acquire resistant determinants and hypervirulent elements, *K. pneumoniae* is now a global threat causing considerable mortality in the affected patients. Evolving gradually from multidrug-resistant (MDR) to extensively drug-resistant (XDR), *K. pneumoniae* isolates had not exhibited simultaneous drug resistance and hypervirulence for long until recently with the emergence of carbapenem-resistant hypervirulent *K. pneumoniae* (CR-hvKP) (2). The primary reason for this delayed emergence has been the successive convergence of two distinct traits of drug resistance and virulence through recombination of several plasmids, resulting in MDR-virulent strains (3). While hvKP has been associated with community-acquired infections, increasing reports of healthcare-associated infections are also being reported (4).

Over the years, hvKP has spread like an epidemic in Asian countries like China, South Korea, and Japan, with the first case being reported from Taiwan in 1986 (5). Widely prevalent and diverse convergent MDR-virulent strains of *K. pneumoniae* have already been reported from the South Asian region including India (6). There have been sporadic reports from India on CR-hvKP in invasive infections revealing the seriousness of the situation. The first report on CR-hvKP in sepsis from the subcontinent came in 2018 followed by a single case of neonatal sepsis recently (7, 8). We report a series of neonatal sepsis due to CR-hvKP along with simultaneous resistance to polymyxins causing high mortality from a tertiary care center.

## Materials and Methods

### Study Site and Design

The study was conducted at the Institute of Medical Sciences, Banaras Hindu University, in Varanasi, and Assam University, Silchar, Assam, India. During a retrospective audit on mortality in the neonatal intensive care unit (NICU), a cluster of cases of neonatal sepsis due to MDR *K. pneumonia* with significant mortality was found during the months of April to June 2017. The isolates were revived from stock cultures and studied as detailed below. The study was approved by the Institute's ethical committee.

### Bacterial Strains and Identification

A total of 9 isolates that were biochemically identified as *K. pneumoniae* were revived by subculture on MacConkey agar and were re-identified by a VITEK® 2 compact system (bioMerieux, USA).

### Antimicrobial Susceptibility Testing

Susceptibility testing was done using both disk diffusion method and VITEK® 2 compact system on the following antibiotics: amoxicillin/clavulanic acid, piperacillin/tazobactam, cefuroxime, ceftriaxone, cefoperazone, cefepime, ertapenem, imipenem, meropenem, amikacin, gentamicin, ciprofloxacin, levofloxacin, and trimethoprim/sulfamethoxazole. For susceptibility against polymyxin B and colistin, broth microdilution was performed. Results were interpreted based on CLSI 2020 guidelines (9). For quality control, *Escherichia coli* ATCC 25922 was used.

### Molecular Characterization of Extended Spectrum β Lactamases, Carbapenemases, and Aminoglycoside-Resistant Determinants

All the isolates were tested for the presence of ESBL genes, as well as carbapenemase and aminoglycoside resistance-encoding genes. Briefly, multiplex PCR was performed for the amplification of ESBL genes (bla_TEM_, bla_SHV_, bla_CTXM1,2,9)_ along with class A (*bla*_SME_, *bla*_NMC_, *bla*_GES_, *bla*_KPC_), class B (*bla*_IMP_, *bla*_VIM_, *bla*_NDM_), and class D (*bla*_OXA_-_48_), carbapenemase genes using primers and reaction conditions as described elsewhere (10, 11). The isolates were further screened for the presence of aminoglycoside resistance genes by three multiplex PCR assays targeting different aminoglycoside resistance genes, viz., *ant(2*″*)-Ia, ant(3*″*)-I, ant(4*′*)-Ia, aac(3)-I, aac(3)-IIc, aac(6*′*)-Ib, aac(6*′*)-II, aph(2*″*)-Ib, aph(2*″*)-Ic, aph(2*″*)-Id, aph(3*′*)-I, aph(3*′*)-IIb, aph(3*′*)-IIIa, aph(3*′*)-VI a*, and *aph(4)-Ia* (12).

### Determination of Hypervirulence Genes in *K. pneumoniae* Isolates

All the isolates were selected for the characterization of different virulence genes by five different multiplex PCR assays. For amplification and characterization of hypervirulence genes, a set of 29 primers was designed in-house using the NCBI primer blast tool (https://www.ncbi.nlm.nih.gov/tools/primer-blast/); from the nucleotide sequence of virulence genes, the amplified products were further sequenced to confirm the presence of virulence genes. The primer sequences and running conditions are summarized in Supplementary Table 1.

### Replicon Typing

For performing PCR-based replicon typing, plasmids from the *K. pneumoniae* isolates were extracted (QIAprep Spin Miniprep Kit, QIAGEN, Germany) and were transformed into *E. coli* JM107. Transformants were selected in the media containing 100 μg/ml of ampicillin. Plasmids isolated from transformants were subjected to PCR assay. Eighteen pairs of primers were used to perform five multiplex and three simplex PCR assays recognizing FIA, FIB, FIC, HI1, HI2, I1-Ig, L/M, N, P, W, T, A/C, K, B/O, X, Y, F, and FIIA (13).

### Multilocus Sequence Typing

Multilocus sequence typing (MLST) was carried out for all *K. pneumoniae* isolates. Genomic DNA was extracted using QIAamp® DNA mini kit as per manufacturer instruction. Amplification of the seven housekeeping genes *rpoB, gapA, mdh, pgi, PhoE, infB*, and *tonB* was done using the primer pairs and conditions as described earlier (14). The amplified product from all nine isolates was sequenced by Sanger sequencing (EzeDiagnon Healthcare PVT., LTD, India). The obtained sequences were assembled by BioEdit v7.2.5 software. The sequence types were determined after analyzing with the help of the Institut Pasteur *K. pneumoniae* MLST database (https://bigsdb.pasteur.fr/klebsiella/klebsiella.html).

### Typing of the Isolates by ERIC-PCR

The heterogeneity of the isolates was determined by enterobacterial repetitive intergenic consensus (ERIC) PCR using the universal primer ERIC-F (5′-ATGTAAGCTCCTGGGGATTCAC-3′) and ERIC-R (5′-AAGTAAGTGACTGGGGTGAGCG-3′). Amplification was done under previously described reaction conditions, and the bands patterns were analyzed by agarose gel electrophoresis (15). Based on the band patterns, the dendrogram was constructed by using computer program NTSYS-pc version 2.0.

## Results

A total of 9 isolates of *K. pneumoniae* subspecies *pneumoniae* were characterized. The characteristics of the patients who were the source of these isolates are shown in Table 1. Majority of the neonates were of very low birth weight (<1,500 g, 66.6%), were pre-term/pre-mature (55.5%), and presented with respiratory distress (6, 66.6%). All but 1 (88.8%) mother of these cases of neonatal sepsis had complications during or prior to delivery in form of preeclampsia, heart disease, and abnormal vaginal discharges. In majority of the cases (6, 66.6%), the mode of delivery was by lower-segment Cesarean section (LSCS). All the cases were empirically treated with piperacillin–tazobactam and amikacin followed by imipenem/meropenem and vancomycin. As a last resort, treatment with polymyxin B was also given. However, all the neonates finally succumbed to the condition (100%).

**Table 1:** Details of neonates and *K. pneumoniae* isolates in the study.

**S. no**.	**Isolate identity**	**Source**	**DoA**	**DoI**	**Risk factors**				
					**Vlbw**	**Respiratory distress**	**Pre-term/pre-mature**	**Complications in mother**	**Mode of delivery**
1	BK1	Blood	20 April 17	24 April 17	N	Y	N	Y	LSCS
2	BK2	Blood	05 April 17	28 April 17	Y	N	Y	Y	LSCS
3	BK3	Blood	27 April 17	01 May 17	N	N	Y	Y	LSCS
4	BK4	Blood	27 April 17	01 May 17	Y	Y	Y	Y	LSCS
5	BK5	Blood	02 May 17	02 May 17	Y	Y	N	Y	SVD
6	BK6	Blood	07 May 17	09 May 17	N	N	Y	N	LSCS
7	BK7	Blood	08 May 17	14 May 17	Y	Y	N	Y	SVD
8	BK9	Blood	21 May 17	24 May 17	Y	Y	N	Y	SVD
9	BK14	Blood	30 May 17	03 June 17	Y	Y	Y	Y	LSCS

*DoA, date of admission; DoI, date of isolation; Vlbw, very low birth weight; N, no; Y, yes; LSCS, lower-segment cesarean section; SVD, spontaneous vaginal delivery*.

All the isolates were extensively drug resistant (XDR) including resistance to polymyxins as per the resistance profile shown in Table 2. The MIC range noted for polymyxin B was 4–32 μg/mL whereas that for colistin was 4–16 μg/mL. Presence of ESBL genes and carbapenemase- and aminoglycoside-encoding genes is shown in Table 3. All the isolates harbored *bla*_SHV_, *bla*_CTXM_, and *aac(6*′*)-Ib*. Among the class B carbapenemases, *bla*_NDM_ and/or *bla*_OXA−48_ were detected in 4 isolates while 5 isolates did not harbor these genes. None of the isolates had class A carbapenemase genes.

**Table 2:** Resistance profile of *K. pneumoniae* isolates.

**Isolates**	**Antimicrobial resistance profile**															
	**AMC**	**PTZ**	**CXM**	**CRO**	**CFP**	**CPM**	**LVX**	**ETP**	**IPM**	**MEM**	**AMK**	**GEN**	**CIP**	**SXT**	CL^*^	PB^*^
BK1	R	R	R	R	R	R	R	R	R	R	I	R	R	S	R	R
BK2	R	R	R	R	R	R	R	R	R	R	R	R	R	R	R	R
BK3	R	R	R	R	R	R	R	R	R	R	R	R	R	R	R	R
BK4	R	R	R	R	R	R	R	R	R	R	I	R	R	S	R	R
BK5	R	R	R	R	R	R	R	R	R	R	R	R	R	R	R	R
BK6	R	R	R	R	R	R	R	R	R	R	R	R	R	R	R	R
BK7	R	R	R	R	R	R	R	R	R	R	R	R	R	R	R	R
BK9	R	R	R	R	R	R	R	R	R	R	R	R	R	R	R	R
BK14	R	R	R	R	R	R	R	R	R	R	R	R	R	R	R	R

*AMC, amoxicillin/clavulanic; PTZ, piperacillin/tazobactam; CXM, cefuroxime; CRO, ceftriaxone; CFP, cefoperazone; CPM, cefepime; LVX, levofloxacin; ETP, ertapenem; IPM, imipenem; MEM, meropenem; AMK, amikacin; GEN, gentamicin; CIP, ciprofloxacin; SXT, trimethoprim/sulfamethoxazole; CL, colistin; PB, polymyxin B; R, resistant; I, intermediate; S, susceptible*.

* *Microbroth dilution method*.

**Table 3:** Summary of *K. pneumoniae* isolates showing resistance genes, ERIC, MLST and plasmid profiles, and virulence genes.

**S. no**	**Isolate identity**	**ERIC type (group)**	**Sequence type (ST)**	**Plasmid**	**AMR genes**	**Virulence genes**			
						**Hypermucoid**	**Iron uptake**	**Allantoin metabolism**	**Siderophores**
1	BK1	A	5235	IncFIC	*bla*_SHV_, *bla*_CTXM_, *bla*_NDM_, *bla*_OXA−48_, *aac(6′)-Ib*	*rmpA2*	-	-	*ybtQ, ybtA, ybtE, ybtS, iroC, irp1, irp2*
2	BK2	B	5235		*bla*_SHV_, *bla*_CTXM_, *aac(6′)-Ib*	*rmpA2*	-	-	*ybtQ, ybtA, ybtE, ybtS, iroC, irp1, irp2*
3	BK3	B	5235	IncFIC	*bla*_SHV_, *bla*_CTXM_, *bla*_NDM_, *bla*_OXA−48_, *aac(6′)-Ib*	*rmpA2*	*fyuA*	-	*ybtQ, ybtA, ybtE, ybtS, irp1, irp2*
4	BK4	C	5235	IncFIC	*bla*_SHV_, *bla*_CTXM_, *bla*_NDM_, *bla*_OXA−48_, *aac(6′)-Ib*	*rmpA2*	*fyuA*	-	*ybtQ, ybtA, ybtE, ybtS, ybtU, ybtXirp1, irp2*
5	BK5	D	5235	IncFIC	*bla*_SHV_, *bla*_CTXM_, *aac(6′)-Ib*	*rmpA2*	*fyuA*	*allB*	*ybtQ, ybtE, ybtS, irp1, irp2*
6	BK6	E	5235	IncFIC	*bla*_SHV_, *bla*_CTXM_, *aac(6′)-Ib*	*rmpA2*	*fyuA*	-	*ybtQ, ybtA, ybtE, ybtS, irp1, irp2*
7	BK7	E	5235	IncFIC	*bla*_SHV_, *bla*_CTXM_, *aac(6′)-Ib*	*rmpA2*	*fyuA*	-	*ybtQ, ybtA, ybtE, ybtS, irp1, irp2*
8	BK9	F	5235	IncFIC	*bla*_SHV_, *bla*_CTXM_, *aac(6′)-Ib*	*rmpA2*	*fyuA*	-	*ybtQ, ybtA, ybtE, ybtS, iroC, irp1, irp2*
9	BK14	F	5235	IncFIC	*bla*_SHV_, *bla*_CTXM_, *bla*_OXA−48_, *aac(6′)-Ib*	*rmpA*	*kfuA, kfuB,*	-	*iucA, irp1, irp2*

Among the hypervirulence genes, *rmpA2* was present in 8 isolates while 1 isolate harbored the *rmpA* gene. Iron uptake-encoding genes (*fyuA*/*kfuA, kfuB*) were present in 7 isolates while the gene for allantoin metabolism (*allB*) was seen in a single isolate. Siderophore-encoding genes (*iroC, ybt, irp, iucA*) were present in all the isolates. The distribution of these hypervirulence genes is shown in Table 3.

**Figure 1:**
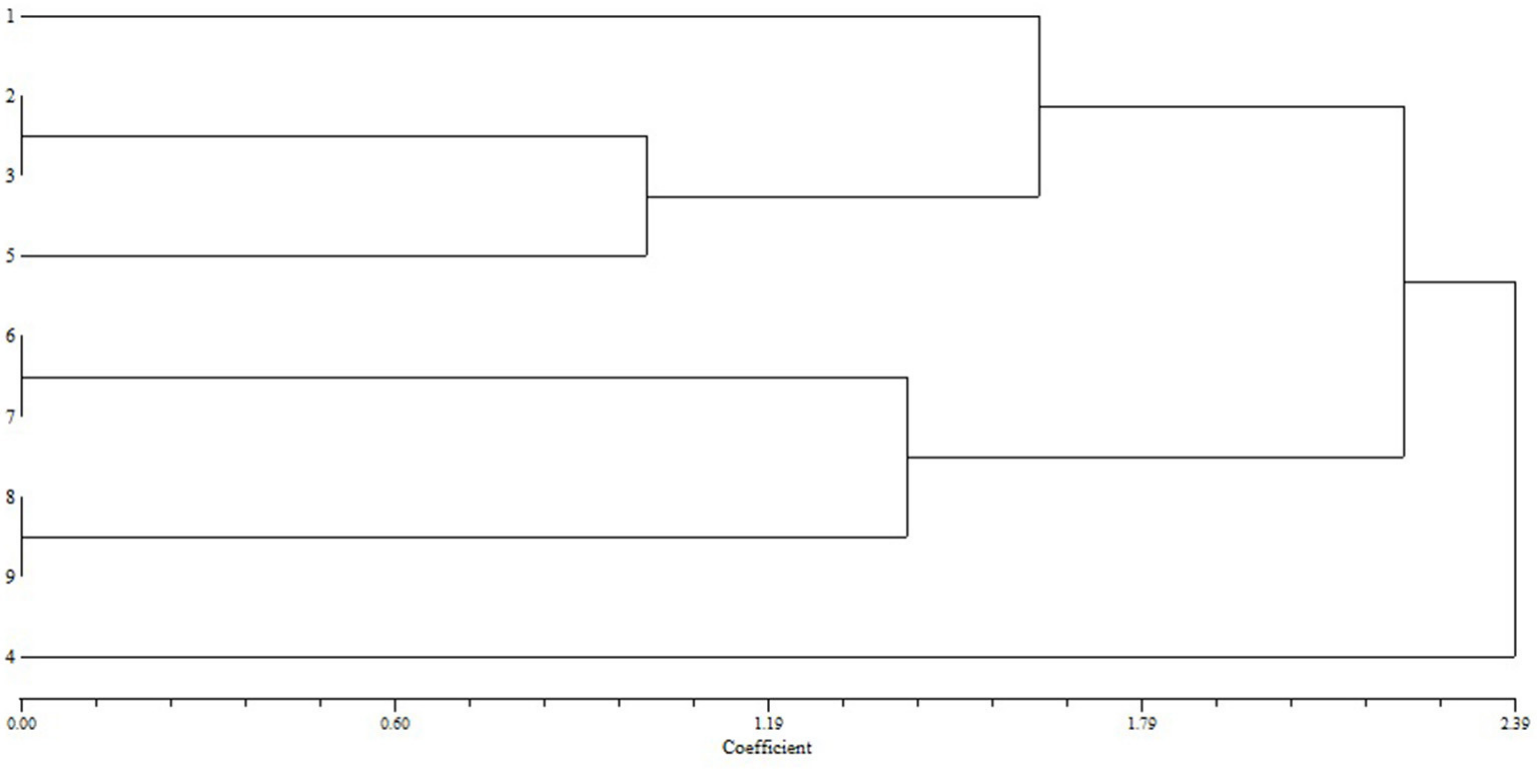
Dendrogram constructed using the ERIC-PCR profile of *K. pneumoniae* isolates.

All the isolates harbored the IncFIc plasmid. Strain typing by ERIC-PCR revealed 100% similarity in 2 isolates each in groups B, E, and F (Figure 1). All the isolates belonged to ST5235.

## Discussion

Carbapenem resistance in *K. pneumoniae* is of critical importance owing to its widespread dissemination. Concurrently, hypervirulence in *K. pneumoniae* is also a serious public health threat. In this study, we report a series of neonatal sepsis due to carbapenem and polymyxin (polymyxin B and colistin)-resistant hvKP causing 100% mortality. To the best of our knowledge, this is the first extensive series of neonatal sepsis caused by XDR-hvKP from the subcontinent.

Hypervirulence has been reported to be associated with very high mortality with rates ranging from 3 to 31%, often increasing up to 35% in case of sepsis (4). This rate further increases when hvKP additionally presents with drug resistance, where the rate as high as 100% has been noted in cases of sepsis due to CR-hvKP (16). Extremely high mortality (84.2%) associated with meropenem-resistant phenotypic and genotypic hvKP-causing sepsis has also been reported from India (6). There has been another single case study on neonatal sepsis from Kolkata, India, with CR-hvKP with inconclusive data on survival as the neonate was lost to follow-up (7). Unfortunately, 100% mortality was seen in the neonates in this study where isolates were also resistant to polymyxins. There have been sporadic reports of colistin-resistant hvKP where isolates retained susceptibility to aminoglycosides, ciprofloxacin, piperacillin–tazobactam, imipenem, and tigecycline despite colistin resistance (17). Consequently, mortality rates were low due to available treatment options.

For long, it was thought and observed that hvKP isolates remained susceptible to antimicrobials until the recent introduction of “convergent” *K. pneumoniae* strains that are both drug resistant and hypervirulent (5). Few of the previous studies have mentioned low drug resistance potential of the hvKP isolates based on either their susceptibilities to the first line of antimicrobials or due to the sporadic presence of ESBL genes not limiting therapeutic options to a greater extent (18). On the other hand, there has been a noticeable upsurge in reports on MDR hvKP especially from developing countries like China, India, Iran, and Brazil where tackling of these isolates has been a big challenge from the treatment point of view (6, 16, 17, 19, 20). The situation is exemplified in the current study where XDR isolates with resistance to last-resort drugs like carbapenems and polymyxins from cases of neonatal sepsis displayed hypervirulence, posing a huge challenge for control. Management of such infections encompasses both adequate source control and active antimicrobial treatment (4). However, it should be mentioned that the neonatal unit in the study followed strict disinfection policy and appropriate infection control practices and even after thorough routine environmental surveillance, no such isolates have been found in the environment or in any of the surveillance cultures from the neonates.

There has been dearth of data and absence of any trial assessing the antimicrobials best suited for treatment of infections caused by hvKP. In contrast, prevalence of MDR in hvKP has been increasing in parallel with the increase in healthcare-associated infections. Suggestions on the use of newer antimicrobial combinations like ceftazidime–avibactam, meropenem–vaborbactam, and imipenem–relebactam for effective treatment of infections by CR-KP have been made (4). However, these antimicrobials have a limited role in infections due to class B carbapenemases, which is one of the major mechanisms of carbapenem resistance in these isolates as revealed in this study and those from developing countries. The *bla*_NDM_ and *bla*_OXA−48_ genes have been often reported to be associated with the hvKP isolates in most of the studies from Asian countries (6, 19). Against antibiotic selection pressure, studies have shown that hvKP isolates can harbor resistance gene plasmids including ESBL, carbapenemases, and those for colistin resistance (5).

It is a well-known fact that several virulence factors of *K. pneumoniae* have facilitated its spread and emergence as a global threat. Among the several genetic markers to define the hypervirulence in *K. pneumoniae*, presence of regulators of the mucoid phenotype, *rmpA* and *rmpA2* genes, produced siderophores like aerobactin, enterobactin, yersiniabactin, and salmochelin, and genes involved in iron uptake and allantoin metabolism are the major ones (18, 21). There has been evidence that *rmpA*/*rmpA2* genes along with siderophores are closely associated with invasive infections (22). All the XDR hvKP isolates in the present study were phenotypically and genotypically hypermucoviscous with multiple types of siderophore-encoding genes. The highly invasive profile of these isolates was reflected in their ability to cause 100% mortality in the neonatal unit.

Emergence of new sequence types among hvKP strains has been noted in few of the recently conducted studies. Though ST23 has been proposed as the major dominant clone of hvKP in Asia (5); most of the infections caused by this clone are community acquired (18). All the XDR hvKP isolates in this study belonged to ST5235, the significance of which could not be discussed owing to paucity of literature on this sequence type.

Despite being an addition to the scarce data on XDR hvKP in neonatal sepsis, this study was not without limitations. While the sources of these isolates could not be traced, classification of the nature of the infections as community or hospital acquired could not be made. Whole-genome sequences of the isolates could have provided better insight into the genome of these isolates. Nevertheless, this study puts forward several yet-to-be-answered issues related to the emergence of XDR hvKP in hospitals. While the origin of these isolates still remains debated, their exact control measures are yet to be determined. More importantly, consensus decisions on optimal therapy against these XDR *K. pneumoniae* isolates with hypervirulence could be the best armament, thus changing the epidemiology of these infections.

## Conclusion

The study analyzed a fatal case series of neonatal sepsis caused by hvKP with extensive drug resistance to carbapenems and polymyxins in a tertiary-care hospital in India, thus revealing the challenges posed by these emerging pathogens in developing countries.

## Data Availability Statement

The datasets generated in this article are not readily available because it is a collaborative study and requires administrative approval for availability. Requests to access the datasets should be directed to ab0404@gmail.com.

## Author Contributions

TB designed the concept and prepared the manuscript. JW performed the experimental work, data collection, and analysis and preparation of the manuscript. SS collected samples and carried out susceptibility profiling. AK helped in clinical analysis of the study. AB supervised the whole work. All authors read and approved the final manuscript.

## Conflict of Interest

The authors declare that the research was conducted in the absence of any commercial or financial relationships that could be construed as a potential conflict of interest.
