# Supplementary material for: Extensively Drug-Resistant Hypervirulent Klebsiella pneumoniae From a Series of Neonatal Sepsis in a Tertiary Care Hospital, India
Source: Front Med (Lausanne). 2021 Mar 8;8:645955. doi: 10.3389/fmed.2021.645955 (PMC7982647; doi:10.3389/fmed.2021.645955)
Supplement: Supplementary file 1 [file Table_1.DOCX]

| **Multiplex PCR** | **Genes** | **Primer sequence** | **Reaction condition** | **Amplified product size (bp)** |
| --- | --- | --- | --- | --- |
| 1^st^ multiplex PCR | iroN-Forward | TTCCTTTCAGGTAGCAGCCG | 95°C-2mins  95°C-20secs  52°C-30secs 35 cycles  72°C-1mins  72°C-5mins | 669bp |
|  | iroN-Reverse | ACCTAGCGAAATCCCGCAAA |  |  |
|  | iutA-Forward | TTATTCGCCACCACGCTCTT |  | 890bp |
|  | iutA-Reverse | AGCACGCCATTCGTCAGTAA |  |  |
|  | iucB-Forward | AGCTAAACGGCCTATCGTCG |  | 368bp |
|  | iucB-Reverse | AAGCGACATCATCCATCGCA |  |  |
|  | iucC-Forward | CAGTCGTTATCCAGCCCGTT |  | 221bp |
|  | iucC-Reverse | AATAAAGGTCGTCGGGGCTG |  |  |
|  | iucD-Forward | GGCGGCATACTGAGATCGAA |  | 751bp |
|  | iucD-Reverse | GCTTCCACGCCAGTGAAATG |  |  |
| 2^nd^ multiplex PCR | rmpA-Forward | CAATGGATGTGGCTTGACGTTT | 95°C-2mins  95°C-20secs  50°C-40secs 35 cycles  72°C-1:20mins  72°C-7mins | 196bp |
|  | rmpA-Reverse | ATTGCAGCACTGCTTGTTCCT |  |  |
|  | rmpA2-Forward | TGAAGGCTCGATGGATAAACC |  | 350bp |
|  | rmpA2-Reverse | CCCTCCTGGAGAGTAAGCATT |  |  |
|  | iroB-Forward | GGTTTTTCGCCACTTCGCTT |  | 562bp |
|  | iroB-Reverse | AACGACGGCGAACCCATTAT |  |  |
|  | iroC-Forward | CGGGAAATAGACGGCAAGGT |  | 978bp |
|  | iroC-Reverse | AGCCGAATTGTGATGCTGGA |  |  |
|  | iroD-Forward | AACCAAGCCCGTCAATCAGT |  | 633bp |
|  | iroD-Reverse | ACGTTGATGTTTTGCAGCCC |  |  |
| 3^rd^ multiplex | iucA-Forward | GGTCACGGCAAAAGTGGAGT | 95°C-2mins  95°C-20secs  51°C-40secs 35 cycles  72°C-1:20mins  72°C-7mins | 600bp |
|  | iucA-Reverse | GAAGTGAAGCGTGGAATGCG |  |  |
|  | entB-Forward | AGTGGTGGCTAATATCGCCG |  | 522bp |
|  | entB-Reverse | TCATCGGATTCGTCGAGCAG |  |  |
|  | allS-Forward | AATGGCGAAGGGGGACGC |  | 807bp |
|  | allS-Reverse | CGATGCTCGATCCCGAAACAT |  |  |
|  | KfuB-Forward | TTGCCGTTAGGGTTTGTGGT |  | 426bp |
|  | KfuB-Reverse | GAAGAACACTTTCCACGGCG |  |  |
|  | KfuC-Forward | TCGAAATCCTTCGCTGATGC |  | 935bp |
|  | KfuC-Reverse | CTCACGGTTCGCAGGACAAT |  |  |
| 4^th^ multiplex | entF-Forward | TATCGATGGTGTGCAGGCAG | 95°C-2mins  95°C-20secs  50°C-40secs 35 cycles  72°C-1:20mins  72°C-7mins | 835bp |
|  | entF-Reverse | GTCGTCGGCAGATAACGGAT |  |  |
|  | allB-Forward | CTGATCGTTTCGCCGGGTAT |  | 528bp |
|  | allB-Reverse | AATGGCTTCCACTTCGGTGA |  |  |
|  | KfuA-Forward | GAAGTCGTGGGTGGATGGAT |  | 643bp |
|  | kfuA-Reverse | ACCGGAAATGCTGACGAAGG |  |  |
|  | ybtA-Forward | CACCGCAAACGCAATCTGAA |  | 474bp |
|  | ybtA-Reverse | CCTGAGCCGTTAAGTGGGTT |  |  |
|  | ybtQ-Forward | CATTGATGCGCTGTTACGGG |  | 256bp |
|  | ybtQ-Reverse | GAGGTTTTCATCCACGCTGC |  |  |
| 5^th^ multiplex | ybtE-Forward | GATAAACGCGCAAATCCGCT | 95°C-2mins  95°C-20secs  50°C-40secs 35 cycles  72°C-1:20mins  72°C-7mins | 954bp |
|  | ybtE-Reverse | GTGAGCGACGATTTTACGCC |  |  |
|  | ybtT-Forward | GCATCTGGTCATGTGCCCTT |  | 684bp |
|  | ybtT-Reverse | TGGCGGACAATCTGCGTAAA |  |  |
|  | ybtU-Forward | GGCGGTGCATGATAAGGCTA |  | 475bp |
|  | ybtU-Reverse | GCCTGTATTGTCGTGCGTTC |  |  |
|  | ybtX-Forward | CGAATGTGAAACCGCTGACG |  | 658bp |
|  | ybtX-Reverse | TGCCGGGTGAGGTGTATTTC |  |  |
| 6^th^ multiplex | ybtP-Forward | GGATACGGCCTGTTGCACTA | 95°C-2mins  95°C-20secs  50°C-40secs 35 cycles  72°C-1:20mins  72°C-7mins | 921bp |
|  | ybtP-Reverse | TTGCCAGAGCCGTTGATACA |  |  |
|  | ybtS-Forward | GATACCGCCCTCAATGGTGA |  | 658bp |
|  | ybtS-Reverse | CATCAAAGCGCGTATCGTCC |  |  |
|  | fyuA-Forward | TACAGTGACCTGACCTGGCA |  | 242bp |
|  | fyuA-Reverse | ACGATGTTGTACCCGGAAGG |  |  |

**Supplementary Table 1:** Primers used for determination of hypervirulence and their running conditions
